# Supplementary material for: Defining a recovery-oriented cascade of care for opioid use disorder: A community-driven, statewide cross-sectional assessment
Source: PLoS Med. 2019 Nov 19;16(11):e1002963. doi: 10.1371/journal.pmed.1002963 (PMC6863520; doi:10.1371/journal.pmed.1002963)
Supplement: Attachment — Submitted filename: Response to Editorial Comments.docx [file pmed.1002963.s003.docx]

**Editorial Comments for PMEDICINE-D-19-02173R2 Resubmission**

**EDITORIAL REQUESTS:**

**Comment 1. Please move the sentence regarding STROBE in boldface at the start of the methods section (and incidentally it does not need to be in boldface) to the end of the paragraph just before table 1 (p.8 of the PDF); e.g. "... prior to this stakeholder engagement process. Our study has been described in line with the Strengthening the Reporting ...**

Response 1: We thank the Editor for this clarification and have moved the sentence and removed the boldface from the text.

**Comment 2. Please make sure that your STROBE checklist refers to items by paragraphs not pages.**

Response 2: We apologize for this oversight from the previous revision and have made the changes as requested. The updated STROBE checklist is now included in the submission.

**FIGURES:**

**Comment 1. Please ensure that all main figures are referenced in the manuscript as Fig 1, Fig 2, etc. (including capitalization), rather than Figure 1, Figure 2, etc. Please note, however, that the file names themselves must not include the space, i.e. Fig1.tif, Fig2.tif, etc.**

**Comment 2. We ask that you please confirm that you either created the following figure panels or have written permission from the copyright holder to publish them under a CC-BY license: Fig 1 and 2 the pictograms.**

Response 2: We confirm that we have created all of the content for the named figures and pictograms, and have updated the author submission form with the following response: “No, we confirm that we have created our own content for each of the figures.” Additionally, the captions include credit to one of the co-authors who created the visualizations, “S1 Fig: Credit: Maxwell Krieger, Brown University”.

**Comment 3: Please note that any written confirmation you send to us must clarify that the copyright holder understands and agrees to the terms of this license. Note that an email confirmation is acceptable.**

Response 2: Not applicable, please see response above.

**METADATA:**

**Comment 1. Please ensure that the CRediT author contributions listed for each co-author are completed in full. To qualify for authorship, each author must meet at least one of the seven core contributions (conceptualization; data curation; formal analysis; investigation; methodology; software; validation), as well as at least one of the writing contributions (original draft preparation; review and editing). Authors may also be noted against any of the remaining contributions, however these alone will not qualify a contributor for authorship.**

**Author Rebecca Boss is noted as contributing to "Writing – review & editing" only, which, as mentioned above, does not qualify them for authorship. Please ensure that the full contributions of each author are acknowledged in the ""Add/Edit/Remove Authors"" section of our submission form. If they do not meet criteria for authorship please let us know as soon as possible by emailing plosmedicine@plos.org.**

Response 2: We apologize for this oversight. Author Rebecca Boss contributed to the conceptualization of the manuscript as well as participated in refining the methodology used. The Author List has been updated accordingly in the submission form.

**Comment 2. The US government employee field is applicable to all authors on the manuscript. Your co-authors, Kimberly Paull, Rebecca Lebeau, Cheyenne Thompson, Tom Coderre and Rebecca Boss, appear to be U.S. government employees. Please therefore revise your answer to this question on the submission form. This will affect the licence under which your article will be published, as all published articles from US government employees have to be published in the Public Domain, rather than our CC-BY license.**

Response 2: We thank the Editor for this clarification, we have updated the submission form to indicate “Yes – One or more authors are employees of the U.S. Government”.

**Comment 3. Please ensure that the funders and grant numbers match between the Financial Disclosure field and the Funding Information tab in your submission form. Note that the funders must be provided in the same order in both places as well. Currently, your grant number RFA-CE15-1501 and funder National Center for Injury Prevention and Control are listed on the Funding Information tab but do not appear in the Financial Disclosure field.**

Response 2: We have updated the funding information so that all fields in the submission form are aligned, and the funding information has been removed from the main manuscript.

The Financial Disclosure section of the form now reads as follows:

JDR and BDLM are supported by the COBRE on Opioids and Overdose funded by the National Institute of General Medical Sciences of the National Institutes of Health under grant number P20GM125507. https://www.nigms.nih.gov/

The funders had no role in study design, data collection and analysis, decision to publish, or preparation of the manuscript.

Funding for Rhode Island’s statewide overdose surveillance website, www.PreventOverdoseRI.org, is provided to the Rhode Island Department of Health from the Centers for Disease Control & Prevention under grant number CDC RFA-CE15-1501. <https://www.cdc.gov/drugoverdose/od2a/index.html>.

The funders had no role in study design, data collection and analysis, decision to publish, or preparation of the manuscript.

**Comment 4. Please edit your data statement so that it is clear to readers where they can access the primary datasets used for this manuscript. Please provide urls and accession numbers if necessary to direct readers to the publicly available survey results (e.g. Stages 0 and 4), and contact information (email or url) for the third party state institutions where readers may apply for access to the aggregated data (Stages 1, 2, and 3). Note that authors may not be the primary contact for data access. Please clarify which of the aggregated data are available at https://preventoverdoseri.org/medication-assisted-therapy/**

Response 2: We have updated our submission form to include the following details:
Aggregated data or publicly available survey results were used whenever possible. Data for Stage 0 are available through the National Survey on Drug Use and Health on the website https://nsduhweb.rti.org.

For Stage 1 we partnered with third party state institutions who provided us with aggregated data, we therefore do not have permission to share anything other than the aggregated estimates that were in the manuscript.

Aggregated data for Stages 2 and 3 are available on the webpage https://preventoverdoseri.org/medication-assisted-therapy/.

For Stage 4 we utilized results from the National Recovery Survey conducted in 2016 by Kelly, Bergman, Hoeppner, Vilsaint, and White (2017). https://www.recoveryanswers.org/media/national-addiction-recovery-study/

**ARTICLE BODY:**

**Comment 1. Please indicate the corresponding author in the byline by placing an asterisk (*) after their affiliation number.**

Response 2: We have indicated the Corresponding Author as described, with an asterisk after their affiliation number.

**Comment 2. Please include the corresponding author's email address on the title page of your manuscript, indicated by an asterisk (*). Only an asterisk and the email address itself are required.**

Response 2: We have updated the Corresponding Author section of the Title Page as described, with an asterisk and email address.

**Comment 3. The name for your co-authors Kim Paull, Maxwell S. Krieger, Ashley L. Buchanan and Josiah D. Rich do not match across the article file and the submission form. Please ensure that all author names match verbatim, including the use of a middle name/initials and any special characters (accents, umlauts, etc.), as correct spelling and formatting of author names is vital for accurate indexing in PubMed.**

Response 2: We apologize for this oversight and have updated the submission form to match the article file.

**Comment 4. Please remove the funding information from your manuscript file. The published version is pulled automatically from the “Financial Disclosure” field in the submission form and must not be duplicated. Please therefore take this opportunity to check that the information in this field is correct and completed in full.**

Response 2: We thank the Editor for this clarification and have removed the funding information from the manuscript file.

**SUPPORTING INFORMATION:**

**Comment 1. Please ensure that all Supporting Information files use the following format verbatim throughout the manuscript (including legends and all in-text citations): S1 Fig, S1 Table, S1 Text, S1 Data, etc. Each file type should start at 1 and increase numerically. Please note, however, that the file names themselves must use an underscore rather than a space, i.e. S1_Fig.tif, S1_Table.xlsx, S1_Text.doc, S1_Data.xlsx, S1_STROBE_Checklist.docx, etc.**

Response 2: We have updated all references to supplemental information accordingly, as well as updated the file names for the submission.

**Comment 2. Please ensure that each supporting information file has a legend at the end of the manuscript file, after the Reference list.**

Response 2: We have added a legend section at the end of the manuscript file, “SUPPORTING INFORMATION”.

**Comment 3. Please note that your submission cannot contain any references to generic "supporting information" files and instead must refer to specific files using our naming convention (S1 Fig, S1 Table, S1 Text, S1 Data, SI STROBE Checklist, etc.)**

Response 2: We confirm that our submission does not contain any references to generic supporting information files. We have updated the reference to the STROBE checklist to the naming convention, as well as all references to the supplemental figures.

**Comment 4. Please replace the page numbers in your STROBE statement with paragraph numbers per section (e.g. "Methods, paragraph 1") since the page numbers of the final published paper may be different from the page numbers in the current manuscript.**

Response 4: We thank the Editor for these clarifications and have updated the STROBE Checklist in the revised submission as directed.
