# Supplementary material for: Defining a recovery-oriented cascade of care for opioid use disorder: A community-driven, statewide cross-sectional assessment
Source: PLoS Med. 2019 Nov 19;16(11):e1002963. doi: 10.1371/journal.pmed.1002963 (PMC6863520; doi:10.1371/journal.pmed.1002963)
Supplement: Attachment — Submitted filename: Response to Reviewer Comments (PMEDICINE-D-19-02173)-FINAL.docx [file pmed.1002963.s002.docx]

**Responses for “Defining a recovery-oriented cascade of care for opioid use disorder: A community-driven, statewide cross-sectional assessment”**

**Editorial Comments**

**Comment #1: In your data statement, are you able to name the “third party state institution”? If so, we would suggest including a contact point or person at this institution for readers who may wish to inquire about obtaining access to the relevant data.**

Response: The third-party state institution cannot be named in the data statement.

**Comment #2: Please restructure your title so that the wording falling after the colon provides a study descriptor, e.g., “a cross-sectional study”.**

Response: The title of the article has been revised to read, “Defining a recovery-oriented cascade of care for opioid use disorder: A community-driven, statewide cross-sectional assessment”.

**Comment #3: Please combine the “methods” and “findings” sub-sections of your abstract. The final sentence of the new combined subsection should summarize the study’s main limitations.**

Response: The methods and findings sub-sections have been combined in the revised abstract (page 2, lines 40-53). The final sentence of the new combined subsection (lines 52-53) summarizes the study’s main limitations, reading:

“Limitations included using a national estimate to identify the number of individuals in recovery due to a lack of available state data sources.”

**Comment #4: After your abstract, you will need to ask you to add a new and accessible “author summary” section in non-identical prose. You may find it helpful to consult one or two recent research papers published in *PLoS Medicine* to get a sense of the preferred style.**

Response: We have added an “Author Summary” to the revised manuscript (pages 4-5).

**Comment #5: Please separate the “Methods” and “Results” sections of your main text.**

Response: We have separated the methods and results section of the main text (page 7 and page 9).

**Comment #6: Early in your methods section, please state whether or not your study had a protocol or prespecified analysis plan, and if so, attach the relevant document as a supplementary file. Please highlight all analyses that were not prespecified.**

Response: In the abstract (page 2, line 44) and methods section (page 7, line 162) of the revised manuscript, we have noted that the adaptation and definition of the proposed cascade of care occurred through an iterative stakeholder engagement process. As such, none of the analyses discussed in the manuscript were prespecified.

**Comment #7: Please add a completed checklist for the most appropriate reporting guidelines (which we suspect may be STROBE or RECORD) as a supplementary document and refer to this early in the methods section. In the checklist, please refer to individual elements by section (e.g., “Methods”) and paragraph number rather than by line or page numbers, as the latter generally change in the event of publication.**

Response: The revised submission includes the completed checklist consistent with the Strengthening the Reporting of Observational Studies in Epidemiology (STROBE) Statement as a supplementary document.

**Comment #8: To your methods section, please add a statement about ethics approval, if only to note that approval was judged unnecessary given the data sources.**

Response: We have added an ethics statement to page 7, lines 158-159.

“These evaluation activities did not meet the federal definition of research and, as such, ethics approval was not required.”

**Comment #9: Are you able to add a table giving summary demographic (and relevant clinical and treatment) details for participants, early in your results section? If so, please also add a sentence on said demographic details to your abstract.**

Response: Demographic characteristics are not available for the aggregate estimates reported.

**Comment #10: Please adapt all reference call-outs in your main text to the following style “…treatment interventions [1].” Where multiple citations are made, please ensure that the square brackets contain no spaces.**

Response: All citations have been updated to meet the formatting style of *PLoS Medicine*.

**Comment #11: Please remove all instances of “[Internet]” from your reference list.**

Response: All references have been updated to meet the formatting style of *PLoS Medicine*.

**Reviewer #1**

**Comment #1: First, it can’t be both stages and a continuum. A continuum implies continuity. Stages deny that continuity.**

Response: We thank the reviewer for this comment. In the revised manuscript, we have revised the manuscript to refer to the proposed framework as a “cascade” rather than a “continuum”.

**Comment #2: Second, the authors did not show any evidence that people move from stage to stage, much less that they do so sequentially. Certainly, people could go from Stage 0 to Stage 4 without the intermediate stages.**

Response: In the limitations section (page 20), we acknowledge that the number of individuals included in Stage 4 could be impacted by the definition of recovery used in the National Recovery Survey in several ways. First, this definition could include people who were included in Stage 0 and progressed to Stage 4 without being included in the intermediate stages because they either would not have met diagnostic criteria for opioid use disorder, or used other non-pharmacological treatment modalities. Moreover, in the revised manuscript, we attempted to estimate the number of individuals who achieved recovery from a problem with opioids using the two medications included in the intermediate stages to improve the continuity of definitions used across stages (Hoffman, Vilsaint, Kelly, 2019). Further, some individuals who have resolved a problem with opioids may not adopt an identity as an individual in recovery (Kelly, Abry, Milligan, Bergman, & Hoeppner, 2018) and, as such, broader terms may be necessary to fully capture this population. Finally, we have revised the terminology throughout the manuscript to avoid connotations that we measured individual transitions from stage to stage. As described on pages 15 and 20, this work represented a preliminary, cross-sectional assessment of the OUD care cascade in Rhode Island. Future work will evaluate individual-level trajectories and transitions between stages and improved linkages across datasets, and the following text has been added to the limitations (page 20, lines 446-449):

“Future analyses will focus on improved linkages across datasets that account for movement between the outlying stages (Stages 0 and 4), the system of care transition stages (Stages 1, 2, and 3), and the time-varying nature of the transition of individual patients from one stage to the next.”

Kelly JF, Abry AW, Milligan CM, Bergman BG, Hoeppner BB. On being “in recovery”: A national study of prevalence and correlates of adopting or not adopting a recovery identity among individuals resolving drug and alcohol problems. Psychol Addict Behav. 2018; 32(6): 595–604.

Hoffman LA, Vilsaint C, Kelly JF. Recovery From Opioid Problems in the US Population: Prevalence, Pathways, and Psychological Well-Being. J Addiction Med. 2019; e-pub ahead of print.

**Comment #3: Third, stages, to me, implies exclusively, but here everyone is at Stage 0 and some people are also at Stage 1, 2, 3, or 4.**

Response: The inclusion of Stage 0 is intended to provide a common denominator for all subsequent stages and estimate the scope of the health problem in the health population. All subsequent stages are exclusive. For example, Stage 1 includes individuals who have been diagnosed with OUD while Stage 2 includes those from Stage 1 who have established engagement in medication-based treatment. This has been further clarified in the results, page 15, lines 317-320.

**Comment #4: Fourth, the authors haven’t really shown evidence that these stages exist – they certainly *might* exist and they make some sense, but why these particular definitions?**

Response: The framework proposed by Williams, Nunes, Bisaga, Levin, and Olfson (2019) served as the base of the adapted Rhode Island Cascade of Care for Opioid Use Disorder described in the current submission. This original framework was developed through a systematic review of established metrics of health care access and quality developed by the National Quality Forum and the Agency for Healthcare Research and Quality (Williams et al., 2018) (page 6, lines 133-135). As such, the definitions of each of the stages are based on these metrics that have documented associations with improved health outcomes and represent practical targets for intervention aimed at improving these outcomes (Williams et al., 2019) (see the revised manuscript, page 8, 172-182).

Williams AR, Nunes EV, Bisaga A, Pincus HA, Johnson KA, Campbell AN, Remien RH, Crystal S, Friedmann PD, Levin FR, Olfson M. Developing an opioid use disorder treatment cascade: A review of quality measures. J Subst Abuse Treat. 2018; 91: 57–68.

Williams AR, Nunes EV, Bisaga A, Levin FR, Olfson M. Developing a cascade of care for responding to the opioid epidemic. Am J Drug Alcohol Abuse. 2019; 45(1): 1–10.

**Comment #5: Fifth, many other people are at risk and risk isn’t a yes/no variable. Different people are at different degrees of risk.**

Response: We thank the reviewer for this comment. We recognize that there is significant heterogeneity among those at risk for OUD. To identify the scope of the health problem in the population of Rhode Island, our stakeholder group chose among the broadest definitions of individuals at risk for OUD. In the limitations section of the revised manuscript, we now note that many individuals in this stage may never experience symptoms of OUD and, as such, may never progress to Stage 1 (page 20, lines 439-446). The stakeholder group felt that it was necessary to include this group to integrate the current primary and secondary prevention efforts directed towards this population in an attempt to reduce drug-related harms with current treatment-related initiatives that are directed towards those who progress to the later stages of the cascade and support recovery. We discuss these prevention approaches for persons in stage 0 on page 12, lines 249-261.

**Comment #6: (Page 7) It’s not clear that anything in here is achievable. I certainly hope it is, but no evidence is presented.**

Response: We respectfully disagree with this suggestion. Extensive systematic reviews have shown that use of methadone and buprenorphine (Stage 2) are associated with continuous engagement in treatment (Stage 3) as well as several markers of recovery (Stage 4) such as reductions in opioid use, decreased cravings, and improved social functioning (Bart, 2012). As such, we believe that it is possible that individuals who use heroin or misuse prescription opioids who experience OUD can successfully progress through the cascade of care by engaging in treatment and achieving recovery.

Bart G. Maintenance medication for opiate addiction: The foundation of recovery. J Addict Dis. 2012; 31(3): 207–225.

**Comment #7: (Page 7) You do not want people moving from Stage 0 to Stage 1.**

Response: As discussed in our response to comment #5 above, stage 0 represents the broadest definition of individuals at risk for OUD (i.e., those who have used heroin or misused a prescription opioid in the past 12 months). In the limitations section of the revised manuscript, we now note that many individuals in this stage may never experience symptoms of OUD and, as such, may never progress to Stage 1 (page 20, lines 449-453). In ideal circumstances, no individuals would progress from using a drug to experiencing any harms associated with their use, but the stakeholder group felt that it was necessary to include this group so that primary and secondary prevention efforts directed towards this population are integrated across systems of care with treatment-related efforts. In the revised manuscript, we have clarified that this is a goal of the cascade (see page 11, lines 231-237).

**Comment #8: (Page 7) There is a typo of Stage 5.**

Response: We have corrected this error in the revised manuscript.

**Comment #9: (Page 9) Focusing solely on MAT isn’t a good idea. Saying that you are doing so because the data are available is a bit like the famous drunk who looked for his keys under the lamppost rather than where he dropped them, because the light was better there.**

Response: We respectfully disagree with this suggestion. The use of medications for opioid use disorder (MOUD), including methadone and buprenorphine, significantly improve OUD outcomes. Use of these medications are associated with reduced risk of death due to opioid overdose, transmission of blood-borne infections, and criminal behavior and are more cost-effective than treatment with no medication and no treatment at all (Blanco & Volkow, 2019). However, experts have noted that their effectiveness is limited by problems at all levels of the cascade of care, including infrequent diagnosis, low rates of entry into treatment, and retention in treatment (Blanco & Volkow, 2019). As such, we believe that the focus on MOUD as the gold standard approach for treating OUD is both appropriate and justified. Nonetheless, in the limitations of the revised manuscript, we acknowledge that individuals may use other non-pharmacological treatment modalities to achieve recovery and may progress from Stage 0 to Stage 4 without progressing through the intermediate stages (page 20, lines 438-444).

Blanco C, Volkow ND. Management of opioid use disorder in the USA: Present status and future directions. Lancet. 2019; 393(10182): 1760–1772.

**Comment #10: (Page 11) For Stage 0, what about people who deny use of opioids? Maybe Stage 0 should be “denial”.**

Response: We recognize that there are limitations inherent in using self-reported measures of heroin use and prescription opioid misuse, including social desirability bias, where individuals who use heroin or misuse prescription opioids may report not doing so to avoid perceived judgement. We have included this as a limitation on page 20, lines 441-444.

**Comment #11: (Page 17) It is not really acceptable that Stage 2 and 3 are defined so differently.**

- Response: We recognize these data limitations, which reflected a statewide challenge in assessing continuous engagement with buprenorphine in office-based settings. Since the first submission, we have worked closely with state agency partners to create better estimates for the cascade. We have updated our results accordingly and have clarified that Stage 3 now includes both methadone and buprenorphine therapy (page 14, Figure 2; page 16, lines 342-349).

The updated text now reads:

“Identifying the number of individuals in Stage 3 also utilized data from the Rhode Island PDMP and BHOLD databases [46,47]. Using these datasets, we identified unique individuals who were engaged with MOUD through a certified opioid treatment program (OTP) or prescribed buprenorphine for at least 180 days without a gap of more than seven days based on the date of first service. Individuals meeting the criteria for retention at any point in 2016 were included.”

**Reviewer #2**

**Comment #1: First, unlike the cascade of care for HIV, to which the paper refers frequently, the stages defined for this cascade do not each represent a closed cohort. This is due largely to the definition of Stage 0 and how it is populated. As far as I can tell, there is no relationship between the dataset used for Stage 0 and the dataset used for Stage 1. There is, therefore, no reason that the population achieving Stage 1 should overlap to any particular extent with the population defined in Stage 0. These could be entirely different people. It’s likely that there is, in fact, a fair bit of overlap, but it’s certainly possible that Stage 1 combines some people identified in Stage 0 and some newly identified at Stage 1. More important, as the manuscript states, many if not most people in Stage 0 will never been eligible for Stage 1. Dropoff between Stages 0 and 1 is thus not meaningful. That is not how a traditional cascade of care works – you normally start with a fixed denominator in the first stage and report proportions achieving each stage after that, with the goal of 100% moving from stage to stage. In this cascade, Stage 0 is problematic, and it tells us little or nothing about retention in care (since most people in it do not start care, and many not in it do start care). While measuring risk in the population is certainly important, it does not belong in this cascade of care, given the nature of OUD and the data available. I recommend removing Stage 0 and starting with Stage 1. A separate presentation of the proportion at risk whom you estimate reach Stage 1 would be fine.**

Response: We thank the reviewer for these comments on Stage 0 of the proposed cascade of care.

We recognize that, as currently defined, the proposed cascade of care does not represent a closed cohort, but this is not uncommon in other cascades of care. Similar to the continuum of care for HIV pre-exposure prophylaxis (PrEP) proposed by Nunn and colleagues (2017), the number of individuals eligible to progress through the cascade of care is not time-fixed. Individuals may be indicated for HIV PrEP use at some points during a given year but not at others given the time-varying nature of risk for HIV infection. Similarly, individuals may be at risk for OUD at some points but not at risk at others given that both relapse and recovery are possible for people living with OUD.

Although the datasets populating Stage 0 and Stage 1 are not linked directly, all individuals diagnosed with OUD in Stage 1 must, by definition, arise from the population of individuals who use heroin or misuse prescription opioids in Stage 0. Nonetheless, we recognize that not all individuals in Stage 0 will progress to Stage 1. At the national level, an estimated 47.5% of individuals who used heroin or misused a prescription opioid in the past 12 months in the 2016 cycle of the National Survey on Drug Use and Health reported at least one symptom included in the formal diagnostic criteria for OUD, suggesting that a substantial proportion of individuals who are included in Stage 0 have the potential to progress to Stage 1 and be diagnosed with OUD. As described in our responses to Reviewer #1, the stakeholder group felt that Stage 0 should be included as a measure of the scope of the health problem in the population and as a first step in efforts to align primary and secondary prevention efforts targeted to this population with treatment-related efforts associated with the later stages of the cascade of care that support achievement of recovery (page 12, lines 245-256).

Nunn AS, Brinkley-Rubinstein L, Oldenburg CE, Mayer KH, Mimiaga MJ, Patel RR, Chan PA. Defining the HIV pre-exposure prophylaxis care continuum. AIDS. 2017; 31(5): 731–734.

**Comment #2: There is no explanation of what we should expect from an OUD cascade of care. What proportions of patients are expected to reach each stage, based on what we know about treatment effectiveness and patient behavior? For non-experts, this information would place the results in context and allow interpretation.**

Response: We thank the reviewer for these comments. In regards to the proportions reached at each stage, the Williams et al. (2019) cascade does include a projection of 90-90-90 targets similar to those of the HIV Cascade of Care. The Rhode Island Cascade of Care represented an initial process to define and populate the cascade for use as a tool to inform next steps. We feel strongly that determining targets for each stage would be a later step in the process, a step that is informed by the current data but led by state leaders and policymakers. On page 9, lines 182-190 now highlight the ways in which an annual snapshot of the cascade can inform potential next steps, including setting targets and standards for improving linkages across stages of care.

Williams AR, Nunes EV, Bisaga A, Levin FR, Olfson M. Developing a cascade of care for responding to the opioid epidemic. Am J Drug Alcohol Abuse. 2019; 45(1): 1–10.

**Comment #3: While the concept of measuring program effectiveness and identifying gaps using a cascade is widely accepted and can be a valuable tool, populating it with unrelated cross-sectional datasets is problematic. Some of the datasets, such as claims data for medication, are probably quite accurate. Others, such as self-reported risk factors and self-reported recovery, are probably not. Moreover, some of the datasets represent national samples and others are state level. The authors mention these limitations but make no effort to consider the potential effect. At the very least, sensitivity analysis to consider the impact of higher and lower rates of achievement of each stage is needed. Ideally, we’ll one day have a prospective cohort that can follow individuals through the cascade; for now, it should be strongly emphasized that these are descriptive data and results and should be interpreted very cautiously.**

Response: We thank the Reviewer for this important comment. In the revised manuscript, we have noted the preliminary nature of the estimates for the numbers of individuals in each stage of the proposed cascade of care and have highlighted opportunities for future research that would fine-tune these estimates. Stages 0 through 3 are populated with state-level estimates specific to Rhode Island, while Stage 4 is populated with an estimate based on a national dataset. Moreover, in the revised manuscript, we have added 95% confidence intervals around the estimates for these stages to show uncertainty around them (Page 14, Figure 2). Finally, we have added a statement on page 20 (lines 449-451) that the descriptive data and results should be interpreted with caution.

**Comment #4: I congratulate the authors on reporting proportions achieving each stage using the original denominator (Stage 0, though I would use Stage 1, as noted above). This is not done in HIV very often and can result in wildly misleading interpretations (e.g., reporting 95% virally suppressed, but only of those who know their status and started treatment and had a viral load test – which is almost a guarantee of suppression – when in fact only half of the infected population knows its status). That said, I think that this manuscript would benefit from including both – the proportion based on the original denominator and the proportion of those reaching the previous stage. The utility of the latter is that it does identify where we are losing people in the cascade. Figure 2 does not emphasize that the proportion lost between Stage 1 and Stage 2 is the most important point of dropoff and should thus likely be the main focus of intervention.**

Response: We thank the reviewer for these comments. In the Results section of the revised manuscript, we have provided proportions of individuals in each stage as a function of the number of individuals included in Stage 0 and as a function of the number of individuals included in the prior stage (lines 306-362).

**Comment #5: The explanation of where in the cascade people go who start treatment but drop off before 7 days is unclear.**

Response: In the revised manuscript, we have clarified that national quality metrics suggest that individuals who initiate MOUD but do not continue to do so for seven days remain in Stage 1 and are not eligible for inclusion in Stage 2 (see page 12, lines 261-266). We also clarified that our definition of Stage 2 was expanded to include any unique individuals who initiated MOUD.

“Stage 2 represents those unique individuals who have “initiated” MOUD treatment. The national quality metrics suggest including only those individuals who have been active on MOUD for more than seven days at one time (representing stabilization) but have been engaged for less than 180 days [29,30]; all others would remain in Stage 1. Nevertheless, the goal for this cascade is to track engagement in care, and therefore we expanded our definition to include any unique individuals who initiated MOUD in 2016.”

**Comment #6: The description of the role of the community stakeholders is also a little unclear. I get that it was due to the community that a final stage of recovery was defined as it was, but there is little explanation in the methods of who participated or exactly what they contributed.**

Response: In the revised manuscript, we have added text to describe the methods used for stakeholder engagement under the heading of “Stakeholder Engagement” (Page 7) prior to discussing the results of the stakeholder engagement process under the heading of “Results” (Pages 9-17).

**Comment #7: Minor, but still important, the writing in this manuscript is sloppy. Many sentences are redundant and there must be a hundred excess commas. There is no clear “Results” section. A good copyeditor is needed.**

Response: We thank the reviewer for this comment. We have carefully reviewed the manuscript to correct typographical errors and ensure clarity and readability of the revised manuscript.

**Comment #8: The title of the article is misleading. The focus is not on identifying gaps, but on defining the cascade and populating it.**

Response: The title of the article has been revised to read, “Defining a recovery-oriented cascade of care for opioid use disorder: A community-driven, statewide cross-sectional assessment”

**Comment #9: To end on a high note, the last sentence in the first paragraph headed “Defining each stage of the RI OUD care continuum” on Page 8, which lists three purposes of the continuum, is really nice. If the paper could be structured around these three goals, it would be stronger.**

Response: We thank the reviewer for these comments. We have restructured the manuscript significantly and we have changed the title of the manuscript to better align with our stated goals for the cascade.

**Reviewer #3**

**Comment #1: It is a strength of the analysis that the authors include a Stage 4 and define recovery broadly, but there are inconsistencies in the way Stage 4 is presented in the paper that are confusing. In the methods section on Page 10, Stage 4 is defined in several ways, including both self-reported recovery (through any possible supportive pathway) and also sustained retention (i.e., >180 days) in medication treatment. This is confusing because Stage 3 is also defined by >180 days in treatment without significant interruption. In the analysis, recovery is only defined by self-report from a national survey. It seems that there are opportunities to use administrative data, including those with sustained uninterrupted retention in treatment, to include people on medications in Stage 4, as the authors suggest is possible in the methods section. Similarly, in the discussion section of Stage 4 on Page 18, the authors omit consideration of ways to include sustained retention as part of Stage 4.**

Response: We thank the reviewer for this comment. In the revised manuscript, we clarify our definition of recovery used and have refined the estimate for recovery to address this concern (page 13, lines 274-284). In the limitations, we acknowledge that the number of individuals included in Stage 4 could be impacted by the definition of recovery used in the National Recovery Survey in several ways. First, this definition could include people who were included in Stage 0 and progressed to Stage 4 without being included in the intermediate stages because they either would not have met diagnostic criteria for OUD or used other non-pharmacological treatment modalities. In the revised manuscript, we attempted to better estimate the number of individuals who achieved recovery from a problem with opioids using the two medications included in the intermediate stages to improve the continuity of definitions used across stages. The results section and figure has been updated accordingly. Further, some individuals who have resolved a problem with opioids may not adopt an identity as an individual in recovery (Kelly, Abry, Milligan, Bergman, & Hoeppner, 2018) and, as such, we note that broader terms may be necessary (page 20, lines 456-458).

Kelly JF, Abry AW, Milligan CM, Bergman BG, Hoeppner BB. On being “in recovery”: A national study of prevalence and correlates of adopting or not adopting a recovery identity among individuals resolving drug and alcohol problems. Psychol Addict Behav. 2018; 32(6): 595–604.

Hoffman LA, Vilsaint C, Kelly JF. Recovery From Opioid Problems in the US Population: Prevalence, Pathways, and Psychological Well-Being. J Addict Med. 2019; doi:10.1097/ADM.0000000000000561

**Comment #2: There is no consideration of how statistical uncertainty should be incorporated into the estimates presented. While quantifying uncertainty might not be indicated for estimates from a complete sample of the population (e.g., the complete PDMP database), uncertainty estimates should be included around point estimates from probability samples (specifically, the National Survey on Drug Use and Health and the National Recovery Survey).**

Response: In the revised manuscript, we have noted the preliminary nature of the estimates for the numbers of individuals in each stage of the proposed cascade of care and have highlighted opportunities for future research that would fine-tune the estimates for each stage. Stages 0 through 3 are populated with state-level estimates specific to Rhode Island, while Stage 4 is populated with an estimate based on a national dataset. In the revised manuscript, we have also added 95% confidence intervals around the estimates for Stage 0 and Stage 4 to show uncertainty around them (page 14, Figure 2).

**Comment #3: A major limitation of the analysis is the exclusion of people retained in buprenorphine treatment from Stage 3, though this limitation is highlighted and discussed in the manuscript. The authors discuss how gaps between prescriptions exclude many people on buprenorphine from meeting the criteria for Stage 3 and they present the mean number of days between prescriptions and average number of refills per year. It would also be more instructive to also present the number of people on buprenorphine who met criteria for Stage 3 (no treatment interruptions >7 days or some equivalent formulation based on number of fills) and the number that did not. The authors could then justify whether or not to include these people in the total for Stage 3.**

Response: We thank the reviewer for this concern regarding the analysis. As discussed in response to Reviewer 1, we recognize these data limitations, and agree they must be resolved. Obtaining results for buprenorphine retention reflected a statewide challenge in assessing continuous engagement with buprenorphine in office-based settings. We have continued to work closely with state agency partners to estimate preliminary estimates for buprenorphine retention >180 days for Stage 3 using the statewide Prescription Drug Monitoring Database (PDMP) data. The total number of people who had a prescription filled for buprenorphine (any version/code used for OUD) for >180 days, with no gaps >7 days was approximately 2,700 people. We have updated our results accordingly (page 14, Figure 2), and have clarified in the manuscript that Stage 3 now includes 2016 retention numbers for both methadone and buprenorphine (page 15, lines 342-349).

**Comment #4: A minor point from the discussion. When stating that RI has high rates of initiation and retention in medication treatment for OUD, it would be helpful to reference what your standard is for determining “high” (e.g., rates from other states).**

Response: We have updated the manuscript with the following detail and citation on Page 17, lines 369-371 to read, “Initial estimates of the number of individuals in each stage indicated that Rhode Island has high initiation and retention rates for engagement in MOUD following a diagnosis with OUD. This is consistent with 2017 data from the National Survey of Substance Abuse Treatment Services which showed that Rhode Island had a rate of 419 per 100 000 people aged 18 years and older engaged in MOUD, ranking among the top five states in the nation [48].”

Substance Abuse and Mental Health Services Administration, National Survey of Substance Abuse Treatment Services (N-SSATS): 2017. Data on Substance Abuse Treatment Facilities. Rockville, MD: Substance Abuse and Mental Health Services Administration, 2018.
